# Significant Shifts in Microbial Communities Associated with Scleractinian Corals in Response to Algae Overgrowth

**DOI:** 10.3390/microorganisms10112196

**Published:** 2022-11-05

**Authors:** Chunrong Lu, Qi Zhang, Qinyu Huang, Shuying Wang, Xiao Qin, Tianfei Ren, Rufeng Xie, Hongfei Su

**Affiliations:** 1 Coral Reef Research Center of China, Guangxi Laboratory on the Study of Coral Reefs in the South China Sea, School of Marine Sciences, Guangxi University, Nanning 530004, China; 2 School of Resources, Environment and Materials, Guangxi University, Nanning 530004, China

**Keywords:** microbial communities, scleractinian corals, benthic algae, coral–algal interactions, 16S rRNA

## Abstract

Microbes play a key role in reef dynamics, mediating the competition between scleractinian corals and benthic algae; however, major shifts in bacterial communities among coral species in response to increases in the abundance of algae are not well understood. We investigated the taxonomic composition of coral-associated microbial communities under algae-overgrowth conditions using 16S rRNA gene sequencing. The results showed that non-algal (i.e., healthy) tissue (HH) had lower bacterial abundance and diversity than tissue collected from the coral–algae interface boundary (HA) and areas of algae growth (AA). Specifically, the HA and AA samples had higher relative abundances of Saprospiraceae, Rhodobacteraceae, and Alteromonadaceae. Compared with *Platygyra* sp. and *Montipora* sp., the physiological response of *Pocillopora* sp. was more intense under algae-induced stress based on microbial gene function prediction. Our results indicate that algal pressure can significantly alter the microbial community structure and function of coral ecosystems. Our data thus provide new insight into the relationship between corals and their microbiome under environmental stress.

## 1. Introduction

Coral reefs are key components of marine ecosystems because of their high ecological and economic value. Coral reefs are representative ecosystems of tropical oceans; provide habitats for reef organisms; protect the coastal zone from erosion; and provide fishery, tourism, and biodiversity resources [1,2]. Coral reefs only account for less than 0.1% of the ocean floor, but harbor more than 25% of total marine species [3,4]. The consequences of global climate change and anthropogenic interference, such as warming, ocean acidification, and eutrophication, are emerging as causes of coral bleaching and various coral diseases, leading to a severe loss of coral cover [5,6]. Approximately 40 coral diseases have been identified to date, with a continual increase since the first report of coral disease in 1973 [7], causing a 30% decline in the living coral worldwide population in the last few decades [8]. This degradation of coral cover provides an opportunity for benthic algae to grow on coral reefs, causing worldwide overgrowth of benthic algae.

Benthic algae are important members of coral reef ecosystems and among the main primary producers, providing food for numerous reef organisms [9]. In this ecosystem, benthic algae and corals are the main competitors for space [10], and changes in competitive advantage can lead to changes in coral reef communities [11]. Significant growth of benthic algae on the reef is when there is a high nutrient input [12], or because of a decrease in herbivorous organisms such as fish (commonly due to overfishing) [13,14]. In addition, environmental disturbances significantly reduce the competitiveness of corals by inducing coral diseases and bleaching, while enhancing the competitiveness of algae [11,15], leading to a phase shift from coral-dominated to algae-dominated coral reef ecosystems worldwide [16,17]. For example, a large-scale bleaching event in the Seychelles coral reef decreased the coral coverage by more than 90%, whereas the coverage of macroalgae increased by 39% in the same period [18]. Moreover, algae inhibit the settlement and recovery of corals through feedback mechanisms, thereby maintaining or enhancing their competitive advantage [19]. Turf algae cover was shown to significantly inhibit coral recruitment [20], and coral skeletons remained covered with algae for up to 2.5 years [21], reflecting the difficulty of recovery from coral damage. These phase shifts can damage the complexity of reef structures, affect the functioning of coral reef ecosystems, and may eventually result in the large-scale loss of marine biodiversity. In contrast, benthic crustose coralline algae (CCA) play a positive role in coral supplementation and conservation, facilitating the consolidation of many modern coral reefs [22,23,24]. Thus, benthic algae in reefs could change coral communities by inhibiting the ability of coral growth and recruitment; consequently, abnormal growth of algae will affect coral growth and maintenance of coverage, thereby impacting the complexity of reef structures which translates to functional changes of the reef ecosystem at the ecological level. Nevertheless, there are still no effective measures to mitigate the damage of benthic algae to the global reef ecosystem, necessitating further research on coral–algae interactions.

Previous studies have explored the mechanisms of algae–coral interactions, demonstrating that algae directly affect corals via allelopathy, shading, and abrasion, and indirectly affect coral health by altering the composition of symbiotic microorganisms of corals [25,26,27]. Symbiotic microorganisms facilitate the growth, reproduction, and metabolism of coral [28], and are also the main responders to environmental changes in coral communities [29,30]. Given that a disturbance in symbiotic microorganisms may compromise the coral health status [25], understanding the relationship between microbial community patterns and coral–algae competition is essential for coral conservation. Coral–algae competition and interactions are typically explored at the reef, colony, microbial, and molecular scales [11]. At the microbial scale, differences in microbial communities between reef-building corals and multiple types of algae have been reported [11], and changes in the microbial community structure of corals at different distances from algae growth have been noted [27]. However, few studies have explored the microbial community structure and physiological functions of different parts of various coral species according to the health condition with respect to algal overgrowth.

To fill this gap and improve understanding of the changes in coral’s microbiome during algal overgrowth, in the present study we compared the taxonomic composition of coral-associated bacteria sampled from various tissues of three coral genera using 16S rRNA gene sequencing. We further compared the community structure of coral-associated bacteria among the three host genera. These findings can expand the current understanding of the microbiology of these ubiquitous corals to facilitate the development of coral research. Moreover, these findings can provide insight into the mechanisms by which algae overgrowth induces changes in bacterial communities, highlighting the larger-scale ecological implications of algae overgrowth in the reef ecosystem. Finally, demonstrating the changes in coral-associated bacteria under algal pressure from the perspective of multiple coral species will provide a better fundamental understanding of the interaction between the coral host and its associated bacteria.

## 2. Material and Methods

### 2.1. Study Site and Coral Sample Collection

**Figure 1:**
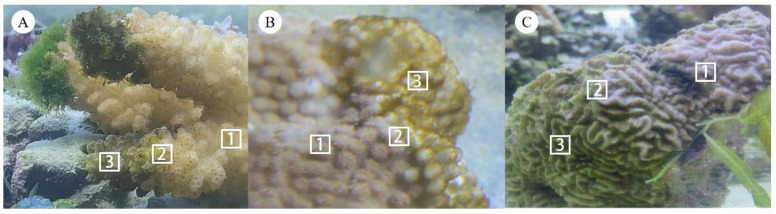
The appearance of algae-growth coral samples: (**A**) *Pocillopora* sp. **(B**) *Montipora* sp. (**C**) *Platygyra* sp. 1, 2 and 3 represented HH, HA and AA samples, respectively.

In April and May 2020, coral fragments (approximately 10 cm × 10 cm) with algae on their surface were sampled, using a hammer and chisel, from 2–10 m depth at Bei Jiao (17°04′48.1″–17°07′3.8″ N, 111°27′00.1″–111°31′34.8″ E), Xisha Islands, China. Three coral species belonging to three genera coexisting in this area were sampled: *Pocillopora* sp., *Montipora* sp., and *Platygyra* sp. To reduce the impact of the shift in environmental conditions, each collected fragment was transported to the laboratory in individual plastic bags containing seawater and immediately placed in a tank set to the same environmental conditions as measured in the sampling area. We obtained three samples from each coral: (1) the non-algae part (HH), which appeared to be healthy on the surface; (2) the algae part (AA), which typically did not comprise coral tissue but only the coral skeleton; and (3) the junction of the non-algae (healthy) part and the algae part (HA). Three replicate samples were collected from each of these three parts on each coral species (Figure 1). The samples were immediately placed in sterile plastic bags and stored at −80 °C until processing.

### 2.2. DNA Extraction, Polymerase Chain Reaction (PCR) Amplification, and Next-Generation Sequencing

Total DNA was extracted from the coral samples (~50 mg) using the MN NucleoSpin 96 Soi Kit (Macherey-Nagel, Düren, Germany), following the manufacturer’s protocol. After extraction and purification, the DNA was used as the PCR template for amplification. The bacterial V3-V4 region of 16S rRNA was amplified using the specific primer pair 335F (5′-CADACTCCTACGGGAGGC-3′) and 769R (5′-ATCCTGTTTGMTMCCCVCRC-3′). The total reaction volume was 10 µL, containing 0.8 µL DNA template, 0.3 µL of each primer, 5 µL KOD FX Neo Buffer, 2 µL dNTP (2 mM each), 0.2 µL KOD FX Neo (Biolink Biotechnology Co., Ltd., Beijing, China), and 1.4 µL ddH_2_O to make up the final volume. The reactions were implemented through the following steps: 95 °C for 5 min; followed by 25 cycles of 94 °C for 30 s, 50 °C for 30 s, and 72 °C for 40 s; and final extension at 72 °C for 7 min. The PCR products were stored at 4°C and then used as templates for Solexa PCR in a total 20 µL volume, including 5 µL PCR template, 2.5 µL MPPI-a (2 µM), 2.5 µL MPPI-b (2 µM), and 10 µL 2 × Q5 High-Fidelity Master Mix. The Solexa PCR was run under the following conditions: 95 °C for 30 s; 10 cycles of 98 °C for 10 s, 65 °C for 30 s, and 72 °C for 30 s; and final extension at 72 °C for 5 min. The products were electrophoresed on an agarose gel (1.8%) at 120 V for 40 min and then mixed at a mass ratio of 1:1 after quantification of the bands using Image J. Mixed DNA products were purified and recovered using a gel extraction kit-V spin column (OMEGA, Shanghai, China). DNA samples were paired-end sequenced (2 × 250 bp) on an Illumina MiSeq platform by Majorbio Bio-Pharm Technology Co., Ltd. (Shanghai, China).

### 2.3. Next-Generation Sequencing Data Processing and Analysis

Raw sequences obtained from each sample were spliced using FLASH (version 1.2.11) [31], and high-quality sequences were obtained after trimming using Trimmomatic (version 0.33) [32] and UCHIME software (version 8.1) [33]. Operational taxonomic units (OTUs) analysis was used to cluster the merged sequences at a 97% similarity level using USEARCH (version 10.0) [34]. The taxonomy of representative sequences was analyzed using the RDP Classifier against the SILVA [35] and Unite (release 128) databases. QIIME software (2020.6.0) was used to generate the species abundance table at different taxonomic levels. OTU-related diversity analysis was performed, including alpha and beta diversity analyses. Alpha diversity refers to the diversity in a specific area or ecosystem, which was calculated using Mothur (version 1.30) [36], including the Chao-1, ACE, Shannon, and Simpson indices. ACE was used to evaluate the richness of the community, in which a higher value represents higher abundance. The Shannon index was used to indicate the diversity of flora, in which a larger index represents higher community diversity. Beta diversity, including principal component analysis (PCA), principal coordinate analysis (PCoA), and sample heatmap analysis, was calculated to analyze changes in species composition on time and space scales using R packages. An unweighted UniFrac method was used to measure the beta diversity of bacterial communities of the three types of corals in the three tissues reflecting different health status (AA, HH, HA). PCA and PCoA results show differences between individuals and groups in the sample. Unweighted pair-group method with arithmetic means (UPGMA) was used for the hierarchical clustering of samples to judge the similarity of species composition among samples. Linear discriminant analysis effect size (LefSe) and Meta-Sat analyses were used to determine the significance of the differences in diversity indices between the groups.

To investigate the differences in the functions of coral-associated microorganisms among different coral species, and the effects of pathogenic bacteria on the overall functions of microbial communities, we used PICRUSt software (version 2.1.0) [37] to predict the composition of functional genes in the samples by comparing species composition obtained from the 16S rRNA sequencing data. PICRUSt2 estimates the functional gene composition of a sample by comparing the abundance of the microbial community with the database, thereby inferring functional information of the microbial community without being observed [38]. Differences in predicted functional categories were evaluated using level 2 of the Kyoto Encyclopedia of Genes and Genomes (KEGG) pathway database.

### 2.4. Statistical Analysis

All data in this study were presented as means ± standard deviation. Student’s *t*-test (parametric test) and Wilcoxon rank-sum test (nonparametric test) were used for comparison between two groups, and analysis of variance (ANOVA, parametric test) and Kruskal–Wallis H test (nonparametric test) was used for comparison among multiple groups. At *p* < 0.05, all differences were considered significant.

## 3. Results

### 3.1. Alpha and Beta Diversity of Coral-Associated Bacteria

A total of 3,020,659 high-quality sequences were obtained from 27 coral samples, with an average of 111,876 sequences per sample. Overall, 106,971–116,333 sequences per sample were obtained, with 108,099–114,913 sequences/sample for *Platygyra* sp., 106,917–116,149 sequences/sample for *Montipora* sp., and 108,886–116,333 sequences/sample for *Pocillopora* sp., with no significant difference among the three coral genera [analysis of variance (ANOVA), *p* = 0.990]. Using Usearch (version 10) [34] to cluster reads at a similarity level of 97.0%, 1526 OTUs were identified, with a maximum number of OTUs in a single sample of 1230. For AA samples, there were 995 OTUs in *Platygyra* sp., 1106 OTUs in *Pocillopora* sp., and 1209 OTUs in *Montipora* sp. For all three genera, the number of OTUs for the three sample types was in the order AA > HA > HH (Table 1). In addition, the Simpson index was showed in Table 1.

**Figure 2:**
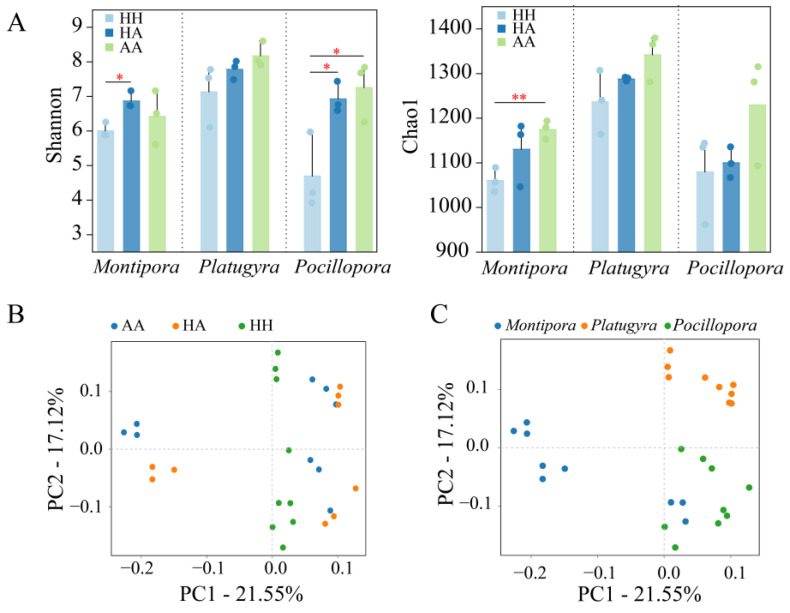
Alpha diversity and beta diversity of three coral genera: (**A**) Shannon index and Chao1 index; *p* < 0.05 and *p* < 0.01 are marked with “*” and “**”, respectively. Unweighted Unifrac PCoA blot showing the differences between bacterial communities based on OTUs among coral health status (**B**) and coral genera (**C**).

The diversity and abundance of coral samples were obtained from the Shannon and Chao1 index, respectively. These indices varied according to the three sample types reflecting the health status for all three coral genera (Figure 2A). The trends in the bacterial diversity among tissue samples were similar for the three coral genera (Figure 2A), with the richness and diversity of the AA samples being significantly higher than those of the HH samples (Student’s *t*-test: Chao1, *p* = 0.020; Shannon, *p* = 0.024). Moreover, the overall Shannon and Chao1 indices were higher for *Platygyra* sp. than for the other two genera.

For beta-diversity, the PCoA results based on the unweighted distance measure showed that the bacterial diversity of the HA and AA samples clustered together, whereas the HH samples were separated from the other two groups (Figure 2B), which was independent of the coral genus (Figure 2C). The UPGMA tree based on unweighted Unifrac distances conformed the result from the PCoA plot (Figure S1). That is, the UPGMA tree showed that the HH samples clustered separately from the other two groups (HA and AA), especially in *Montipora* sp. and *Pocillopora* sp. corals. These results suggest that bacterial diversity changes in competition with algae, and that coral microorganisms may cluster in the same direction or disperse in different directions in response to such competition, which may be related to the intensity of the competition.

### 3.2. Shift in Taxonomic Assignment of Coral-Associated Bacteria

**Figure 3:**
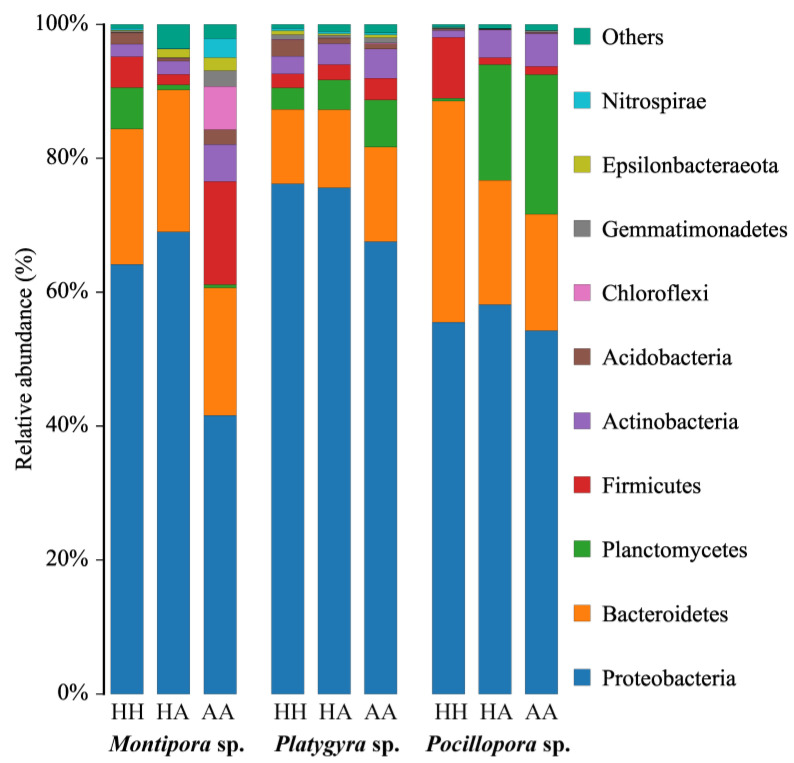
Relative abundance (%) of major bacterial communities at the phylum level.

Based on the SILVA database, the bacterial sequences were classified into 25 phyla, 57 classes, and 427 genera for all samples in taxonomic assignment by QIIME (Table S1). There were some variations in the number of bacterial phyla among the three sample types according to health status for each coral genus. For example, in the case of *Montipora* sp., the bacterial communities in HH samples were dominated by Proteobacteria (64.2%), followed by Bacteroidetes (20.2%), Planctomtomcetes (6.2%), Firmicutes (4.6%), Actinobacteria (1.9%), and Acidobacteria (1.7%), whereas the dominant bacterial communities associated with the AA samples were Proteobacteria (41.3%), followed by Bacteroidetes (19.2%), Firmicutes (15.4%), Chloroflexi (6.5%), Actinobacteria (5.5%), and Nitrospirae (2.8%) (Figure 3). These results suggest that changes in coral health are often accompanied by changes in microbial composition, which is also reflected in other corals. Proteobacteria accounted for 62.4% of the total number of sequences tested and Bacteroidetes accounted for less than 2% of the total number of sequences. Of all samples analyzed, 1.2% were uncategorizable at the phylum level.

At the phylum level, the HH and HA samples had a higher relative abundance of Proteobacteria compared with that of the AA samples for the three coral genera. In *Platygyra* sp. and *Montipora* sp., the relative abundance of Proteobacteria in the AA group was significantly lower than that in the HH group (Student’s *t*-test: *p* = 0.023 and *p* = 0.040, respectively), which was decreased by 22.9% in *Montipora* sp. Actinobacteria showed a relatively small increase in relative abundance in HH compared with that in AA samples for the three coral genera. The relative abundance of Planctomycetes was slightly decreased in *Montipora* sp. from AA to HH samples, but was increased in the other two genera, with a 20.1% increase in *Platygyra* sp. These results suggest that the microbial composition of coral fragments changes to varying degrees depending on the presence of algae growth, which can be correlated with variations among coral species.

**Figure 4:**
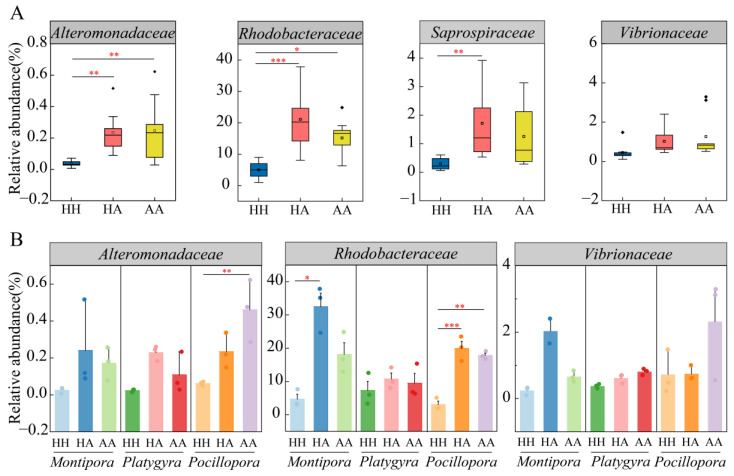
Significant differences in the relative abundance of bacterial categories at the family level: (**A**) Differences between groups; (**B**) Differences between coral genera. *p* < 0.05, *p* < 0.01, and *p* < 0.001 are marked with “*”, “**”, and “***”, respectively.

At a deeper level, the decrease in the relative abundance of Proteobacteria was mainly reflected in changes in Alphaproteobacteria, with a highly significant decreasing trend in relative abundance from the HH samples to the HA and AA samples (ANOVA, *p* = 0.0084). However, less variation was found in the relative abundances of Gammaproteobacteria and Deltaproteobacteria (Figure S2). Alteromonadaceae, Rhodobacteraceae, and Saprospiraceae were the main families that showed significant differences among the groups. For example, the relative abundances of these three families in HH samples were significantly lower than those in HA and AA samples. Among these, the difference between the HH and HA samples was more significant, whereas the compositions of HA and AA samples were similar. Vibrionaceae showed no significant differences among HH, HA, and AA samples (Figure 4A). With respect to variations among coral genera, the changes in Alteromonadaceae and Rhodobacteraceae between sample types were more significant in *Pocillopora* sp. than in the other two genera (Figure 4B). Nevertheless, the variations in relative abundance for Alteromonadaceae, Rhodobacteraceae, and Vibrionaceae showed similar trends in the three coral genera, with higher relative abundance in the HA and AA samples (i.e., in the presence of algae) than in the HH samples (Figure 4B).

At the genus level, pathogenic *Vibrio*, which is associated with multiple epidemic coral diseases, was detected in all coral samples at a relative abundance of 0.13% to 0.60% (Table S2), and no significant decrease in *Vibrio* sp. was observed according to the health status of the tissue (Kruskal–Wallis H test: *p* = 0.132). In addition, *Cyanobacteria* and *Thalassomonas* were not detected in any samples, and a small amount of *Arcobacter* and *Desulfovibrio* was present with a maximum relative abundance of 1.9%, and no significant differences detected among sample types (Kruskal Wallis H test, *Arcobacter*: *p* = 0.927; *Desulfovibrio*: *p* = 0.137) (Table S2).

### 3.3. Microbial Gene Function Variation among Coral Tissues and Species

**Figure 5:**
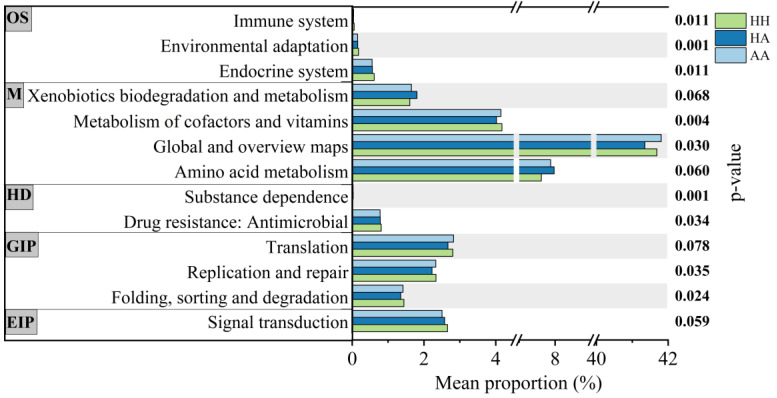
Predicted functional categories in KEGG pathways (level 2) using PICRUSt2. These differences among HH, HA, and AA groups were analyzed using the Kruskal Wallis H test. OS: Organismal Systems, M: Metabolism, HD: Human Diseases, GIP: Genetic Information Processing, and EIP: Environmental Information Processing.

Based on the KEGG database, differences in the function of bacteria were detected among the different samples (Table S3A). Nine of the 44 predicted functional categories showed differences among the HH, HA, and AA groups (Kruskal–Wallis H test, *p* < 0.05; Figure 5), including environmental adaptation, substance dependence, metabolism of cofactors and vitamins, endocrine system, immune system, global and overview maps, folding, sorting and degradation, drug resistance, antimicrobial, and replication and repair (Table S3B). These categories are primarily concentrated in organismal systems, metabolism, human diseases, and genetic information processing. To understand the effect of algal stress on the coral bacterial community, we analyzed the differences between the HH and HA samples of the three coral genera. In *Pocillopora* sp., 30 of the 44 functional categories showed significant differences between the HH and HA groups (Wilcoxon rank-sum test, *p*-value < 0.05, Table S3C), and in *Montipora* sp., 23 categories showed differences between the two groups (Wilcoxon rank-sum test, *p* < 0.05; Table S3C). There were only six different functional categories in *Platygyra* sp., including cellular community–prokaryotes, membrane transport, biosynthesis of other secondary metabolites, metabolism of terpenoids and polyketides, nucleotide metabolism, and excretory system (Wilcoxon rank-sum test, *p* < 0.05; Table S3C). These categories were distributed in cellular processes, environmental information processing, metabolism, and organismal systems.

## 4. Discussion

### 4.1. Algae Overgrowth Significantly Alters the Structure of Coral-Associated Bacterial Communities

After observing that corals were under benthic algal stress, we conducted an in-depth analysis at the molecular level to explore any associated alterations in the relevant bacterial communities. Microbial sequencing analysis confirmed that the bacterial community structure of corals changed significantly in the presence of algae, with significant differences in both diversity and species richness of coral-associated bacterial communities in all three coral genera between the HH and AA samples. Specifically, algal stress on corals led to an increase in bacterial richness and diversity. Bacterial communities are assumed to provide benefits to their hosts, which are closely linked to the growth [39], development [40], and reproduction [28,41] of corals, responding to disturbances in their external environment by changing their community structure [42,43]. Previous studies have shown that healthy corals have a higher diversity of bacterial communities than disease-bearing corals and show higher levels of OTUs [44,45]. However, Pootakham et al. [8] found higher bacterial diversity in corals suffering from bleaching, which is consistent with our findings, suggesting that corals may be stressed by similar diseases when invaded by algae to stimulate a similar response in bacterial communities. Moreover, the changes in diversity and richness were similar across coral genera, although the degree of changes in the bacterial community structure varied across the coral species. These patterns suggest species homogeneity in the factors driving a shift in the trajectories of coral-associated bacterial communities along with a species-specific stress response. The similarities and differences in the microbial structure of corals may be the result of differential interactions between stress factors.

With respect to the bacterial composition, Proteobacteria had the highest relative abundance in all coral samples for all three species, which is consistent with previous related studies [29,46,47]. Moreover, healthy corals harbor a higher relative abundance of Proteobacteria than diseased corals; therefore, bacterial groups belonging to this phylum can serve as an indicator of coral diseases or environmental stress, such as *Vibrio* sp. [48] and Rhodobacteraceae [47]. Moreover, our results revealed that the phylum Proteobacteria was the major cause of differences in microbial abundance detected between the HH and AA samples in *Platygyra* sp. and *Montipora* sp., which is consistent with previous studies on bacterial communities linked to various stress factors [27,47,49]. Furthermore, Alphaproteobacteria was the predominant class of Proteobacteria in these samples.

The relative abundance of the Rhodobacteraceae family, a member of the phylum Proteobacteria, was lower in the HH samples than that in the HA and AA samples, indicating an increase under algae stress. Members of this family are widespread in the marine environment [50,51] and play an important role in the marine biofilm formation process [52]. Rhodobacteraceae show either a competitive or promotive relationship with marine algae; for example, the relative abundance of Rhodobacteraceae generally increases in an algal bloom [53,54], whereas these bacteria mainly compete with algae when external conditions change, such as under a condition of nutrient constraint [53,55]. In coral holobionts, the relative abundance of Rhodobacteraceae typically varies with increasing environmental pressure on corals such as diseases or heat stress [8,56,57]. In addition to the Rhodobacteraceae family, the Alteromonadaceae family, which we found at higher abundance in the interface (HA) and algae growth (AA) coral samples, also belongs to the Proteobacteria phylum, and members of this family have been identified as pathogens of coral disease [49,58]. For example, some bacteria accumulated in *Montastraea faveolata* coral colonies with signs of white plaque disease (WPD) type II were identified as belonging to the Alteromonadaceae family [59]. Furthermore, members of Alteromonadaceae are also common pathogens in aquaculture systems [60], and have been generally linked to coral disease and stress [49].

In general, stress can challenge the stability of the microbial community structure of corals, which may facilitate colonization by various opportunistic bacteria. The detected increase in Rhodobacteraceae and Alteromonadaceae suggests that algal pressure may create favorable conditions for their growth, which may lead to opportunistic colonization. In coral holobionts an imbalance in bacterial composition under algal invasive pressure has been suggested to affect the metabolism of the entire coral organism [61], with potentially adverse consequences for the coral. Interestingly, members of these two families play markedly different roles in algae and coral. Rhodobacteraceae was found to be involved in the induction process of recovering the growth and morphogenesis of the green macroalgal order Ulvales [62], whereas Alteromonadaceae has been associated with algal growth. For example, Yang et al. [63] isolated a faint yellow-pigmented bacterium from the dinoflagellate *Alexandrium catenella* LZT09 that appeared to have potential to promote the growth of its algal host. In corals, members of Rhodobacteraceae were found to scavenge free radicals, which can reduce the oxidative stress-induced damage to coral symbionts [64]. In addition, the family Saprospiraceae, which also showed significant changes in abundance according to algal growth, was proposed as a marker of high temperature, injury, and nitrogen enrichment [65], and has also been found to be enriched in algal disease sites [66].

Moreover, several *Vibrio* species such as *Vibrio shiloi* have been identified as one of the main pathogens in several prevalent coral diseases, including black band disease and WPD [67,68]. We found a relatively low abundance of *Vibrio* in all samples with no significant difference according to the health status of corals, which is consistent with the results of many previous studies on coral disease. For example, Sweet and Bythell [69] detected a relatively low abundance of *Vibrio* species in healthy coral tissues [70] with a similar proportion found in diseased tissue. In addition, *Desulfovibrio* [71], *Roseovarius* [72], and *Cytophaga* [73] are considered to be the main microorganisms that play a pathogenic role in various coral diseases, which were detected in all of the samples tested in this study but at negligible levels, with no significant trends noted according to sample type or coral species. Although all coral samples obtained in this study were clearly facing pressure from overgrowth by algae, the corals showed no signs of common diseases. This suggests that healthy coral tissues may harbor bacterial members that are considered potentially pathogenic [70], highlighting a risk of disease if conditions enable these potential pathogens to reproduce opportunistically.

### 4.2. Algae Overgrowth Impacts the Microbial Function of Corals

The change in coral-associated microorganisms caused a physiological function shift in the coral holobiont, and the change in physiological characteristics of different corals under algal stress differed among the coral genera. Coral microorganisms are important components of the whole coral organism and play a key role in maintaining the normal physiological process of the holobiont [74,75]. Stress such as heat stress affects the stability of coral symbiotic microbial communities and causes changes in physiological characteristics [74,76,77]. For example, Qin et al. (2020) found that physiological characteristics of corals, such as metabolism, genetic information processing, environmental information processing, and cellular processes, were significantly affected during bleaching events, with the most obvious changes found in the metabolism category [76]. However, few studies have reported the response of coral bacterial functions to algal stress. Our functional prediction analysis showed that bacterial functions were significantly different according to coral health status and were also related to coral species. Organismal systems, metabolism, human diseases, and genetic information processing were the main functions affected by algal stress; among these, metabolism was the most strongly affected physiological characteristic. Moreover, by comparing the HH and HA groups, we found that the branching coral *Pocillopora* sp. was more sensitive to algal stress than massive corals (*Montipora* sp. and *Platygyra* sp.), suggesting that changes in the physiological characteristics of coral symbiosis function are similar to those occurring in microbial communities. These effects may be primarily due to the stress of algae overgrowth, as algae are well known to affect coral hosts and their microbial communities from various aspects.

### 4.3. Ecological Implications

The drivers of changes in coral-associated bacterial communities vary, including the effects of primary metabolites, secondary metabolites, and pathogenic bacteria carried by algae. Algae may indirectly affect coral bacterial communities by releasing primary metabolites such as organic carbon to alter coral-associated microbial communities. Increased organic carbon has been reported to play a role in coral death [78], and this negative impact could be prevented or delayed by the treatment of corals with antibiotics [79]. Moreover, microorganisms carried by algae themselves may induce detectable changes in the bacterial community structure of corals. Similar to corals, marine algae harbor diverse microbial communities on their surfaces [70], and these microbes may be transferred to corals by contact or other means, triggering a range of responses from coral symbionts. Algae are reservoirs of many coral pathogens that can be transmitted to corals through contact, causing disease under the synergistic effects of other competitive stresses [45,70]. Moreover, microorganisms carried by algae may induce detectable changes in the coral bacterial community structure, which may at least partly explain the differences in microbial communities found in this study. Algae can also influence various ecological processes of corals, including shading and abrasion [25], although most studies addressing these impacts have focused only on a single factor [80]. In complex ecosystems, the overall impact usually reflects the interaction of multiple factors [70]. Thus, the changes in the microbial community detected in this study may be partly due to the complex interactions and additive effects of multiple factors.

Most natural or anthropogenic disturbances contribute to benthic algae proliferation [81,82] and adversely affect coral ecological processes [83,84], inducing a phase shift from a coral-dominant to algae-dominant community in the coral reef ecosystem [16,17]. Many coral reefs worldwide are facing this transformation, which can result in changes in the structural complexity of coral reef ecosystems, thereby reducing their vital functions. The important tasks in maintaining the balance and vital functions of coral reef ecosystems are increasing coral resilience and decreasing the competitive advantages of benthic algae, which can be achieved through both natural and human-assisted methods. Many studies have shown that corals can be resistant to environmental disturbances and can recover subsequently [18,85,86], depending on the extent and nature of the disturbance. For example, the recovery of corals from macroalgae is faster than that from turf algae and CCA [86]. However, in coral–algae competition, algae overgrowth is closely related to the herbivore’s pressure [13,14,87]. Although algae can secrete chemical toxins to prevent the ingestion of herbivores [88], increasing the diversity of herbivores may be a viable strategy to prevent algae from overgrowing [89]. Therefore, the establishment of protected areas combined with the reduction in human interference can facilitate recovery and reduce the occurrence of new damage. Moreover, the impact of global climate change on coral reefs cannot be ignored, and although the process of mitigating or even reducing the impact of climate change on coral reefs is daunting, ecological conservation strategies should include striving toward a temperate climate.

## 5. Conclusions

Globally, the ecological state and balance of reef ecosystems have been affected by algal overgrowth. In this study of three coral genera, we found that algae overgrowth might cause disturbance to the intricate microbial community of coral, which was reflected by a change in the bacterial diversity and community structure according to the algae cover, along with coral species-specific responses. Further studies examining the changes in coral-associated microorganisms caused by algal overgrowth can provide new insight into the relationship between corals and their microbial communities under stress conditions. This knowledge will be crucial for the restoration of coral reef ecology in the backdrop of widespread degradation.

## Figures and Tables

**Table 1:** Richness and Diversity estimates of bacterial 16S rRNA sequencing data of coral samples. HH: the non-algae part; HA: the junction of the non-algae part and the algae part; AA: the algae part.

Coral Genus	Condition	No. of Replicates	Richness Estimates	Diversity Estimates
			**OTUs**	**Simpson**
			**Mean**	
*Montipora* sp.	HH	3	1048 ± 37	0.94 ±0.00
	HA	3	1196 ± 16	0.97 ± 0.01
	AA	3	1209 ± 21	0.95 ± 0.02
*Platygyra* sp.	HH	3	929 ± 71	0.96 ± 0.02
	HA	3	970 ± 36	0.97 ± 0.01
	AA	3	995 ± 25	0.99 ± 0.00
*Pocillopora* sp.	HH	3	714 ± 91	0.86 ± 0.04
	HA	3	974 ± 19	0.97 ± 0.01
	AA	3	1106 ± 90	0.97 ± 0.01

## Data Availability

Not applicable.

## Supplementary Materials

The following supporting information can be downloaded at: https://www.mdpi.com/article/10.3390/microorganisms10112196/s1, Figure S1 The UPGMA tree of coral samples is based on the Unweighted Unifrac distance matrix. Figure S2 Relative abundance (%) of major bacterial communities at the class level. Table S1 Number of bacteria obtained at all taxonomic levels in 3 coral genera. Table S2 Relative abundance (%) of some important bacterial communities at the genus level. Table S3 The data for KEGG at level 2.
