# Supplementary material for: Significant Shifts in Microbial Communities Associated with Scleractinian Corals in Response to Algae Overgrowth
Source: Microorganisms. 2022 Nov 5;10(11):2196. doi: 10.3390/microorganisms10112196 (PMC9694024; doi:10.3390/microorganisms10112196)
Supplement: Supplementary file 1 [file microorganisms-10-02196-s001.zip › Supplementary material data.pdf]

Figure. S1 The UPGMA tree of coral samples is based on the Unweighted Unifrac distance matrix. A, B, and C represent *Montipora sp*., *Platygyra sp*., and *Pocillopora sp*., respectively. 1,2 and 3 represented HH, HA and AA samples, respectively.

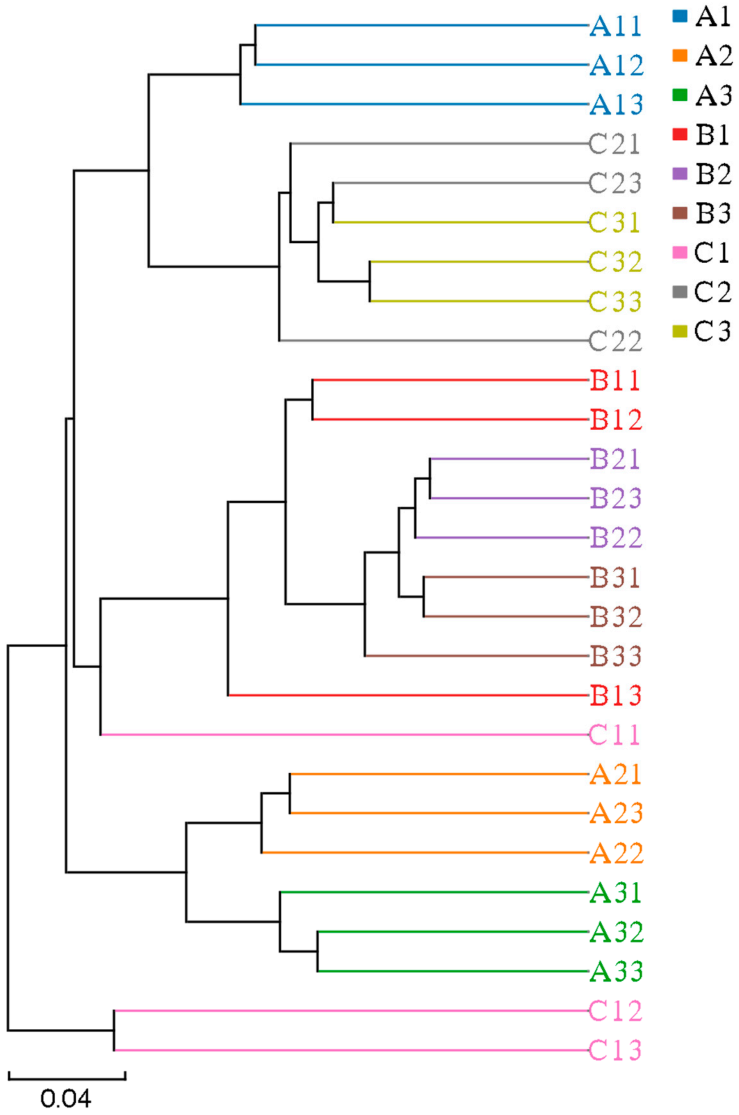

Figure. S2 Relative abundance (%) of major bacterial communities at the class level.

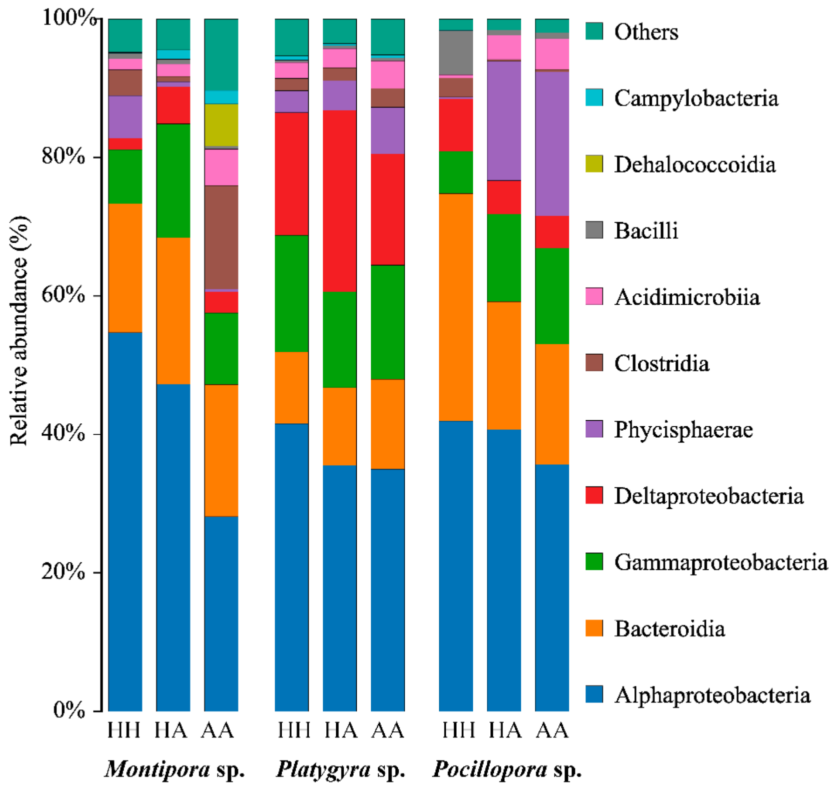

Table S1 Number of bacteria obtained at all taxonomic levels in 3 coral genera

Coral genus	Condition	Phylum	Class	Order	Family	Genus
***Pocillopora* sp.**	HH	23	54	122	221	389
	HD	22	48	115	215	391
	DD	24	53	121	221	397
***Montipora* sp.**	HH	23	49	121	218	382
	HD	23	48	117	212	384
	DD	22	50	118	215	380
***Platygyra* sp.**	HH	23	50	117	213	358
	HD	21	47	116	212	375
	DD	23	50	120	221	396

Table S2 Relative abundance (%) of some important bacterial communities at the genus level.

Coral genus	Condition	Relative abundance (%)		
		Vibrio	***Arcobacter***	***Desulfovibrio***
***Montipora* sp.**	HH	0.123	0.119	0.009
	HA	2.606	1.329	0.003
	AA	0.358	1.984	0.003
***Platygyra* sp.**	HH	0.292	0.230	0.211
	HA	0.401	0.041	0.072
	AA	0.594	0.004	0.101
***Pocillopora* sp.**	HH	0.216	0.018	0.084
	HA	0.127	0.002	0.005
	AA	0.311	0.009	0.004
